# Supplementary material for: Pyruvate Dehydrogenase Kinase 1 inhibition mediated oxidative phosphorylation enhancement in cartilage promotes osteoarthritis progression
Source: BMC Musculoskelet Disord. 2023 Jul 20;24:597. doi: 10.1186/s12891-023-06585-6 (PMC10357736; doi:10.1186/s12891-023-06585-6)
Supplement: Supplementary file 1 — Additional file 1. [file 12891_2023_6585_MOESM1_ESM.zip › KEGG.Gsea.1653622432464/KEGG_NICOTINATE_AND_NICOTINAMIDE_METABOLISM.html]

Details for gene set KEGG\_NICOTINATE\_AND\_NICOTINAMIDE\_METABOLISM[GSEA]

|  || Dataset | 55457\_collapsed\_to\_symbols.cls.cls #OA\_versus\_normal.cls.cls #OA\_versus\_normal\_repos |
| Phenotype | cls.cls#OA\_versus\_normal\_repos |
| Upregulated in class | OA |
| GeneSet | KEGG\_NICOTINATE\_AND\_NICOTINAMIDE\_METABOLISM |
| Enrichment Score (ES) | 0.23316188 |
| Normalized Enrichment Score (NES) | 0.71380836 |
| Nominal p-value | 0.8716707 |
| FDR q-value | 1.0 |
| FWER p-Value | 1.0 |
Table: GSEA Results Summary

  

Fig 1: Enrichment plot: KEGG\_NICOTINATE\_AND\_NICOTINAMIDE\_METABOLISM      
 Profile of the Running ES Score & Positions of GeneSet Members on the Rank Ordered List

  

| SYMBOL | TITLE | RANK IN GENE LIST | RANK METRIC SCORE | RUNNING ES | CORE ENRICHMENT || 1 | NT5C | "5', 3'-nucleotidase, cytosolic [Source:HGNC Symbol;Acc:HGNC:17144]" | 1852 | 0.379 | -0.0625 | Yes |
| 2 | QPRT | quinolinate phosphoribosyltransferase [Source:HGNC Symbol;Acc:HGNC:9755] | 2077 | 0.353 | -0.0042 | Yes |
| 3 | BST1 | bone marrow stromal cell antigen 1 [Source:HGNC Symbol;Acc:HGNC:1118] | 2319 | 0.324 | 0.0466 | Yes |
| 4 | CD38 | CD38 molecule [Source:HGNC Symbol;Acc:HGNC:1667] | 2416 | 0.312 | 0.1061 | Yes |
| 5 | NNT | nicotinamide nucleotide transhydrogenase [Source:HGNC Symbol;Acc:HGNC:7863] | 2425 | 0.311 | 0.1722 | Yes |
| 6 | NMRK1 | nicotinamide riboside kinase 1 [Source:HGNC Symbol;Acc:HGNC:26057] | 2857 | 0.265 | 0.1955 | Yes |
| 7 | ENPP1 | ectonucleotide pyrophosphatase/phosphodiesterase 1 [Source:HGNC Symbol;Acc:HGNC:3356] | 3392 | 0.212 | 0.1996 | Yes |
| 8 | NT5E | 5'-nucleotidase ecto [Source:HGNC Symbol;Acc:HGNC:8021] | 3515 | 0.201 | 0.2332 | Yes |
| 9 | NMNAT2 | nicotinamide nucleotide adenylyltransferase 2 [Source:HGNC Symbol;Acc:HGNC:16789] | 5674 | 0.018 | 0.0694 | No |
| 10 | AOX1 | aldehyde oxidase 1 [Source:HGNC Symbol;Acc:HGNC:553] | 6243 | -0.024 | 0.0304 | No |
| 11 | NT5M | "5',3'-nucleotidase, mitochondrial [Source:HGNC Symbol;Acc:HGNC:15769]" | 7781 | -0.140 | -0.0589 | No |
| 12 | NADSYN1 | NAD synthetase 1 [Source:HGNC Symbol;Acc:HGNC:29832] | 8858 | -0.222 | -0.0949 | No |
| 13 | NT5C2 | "5'-nucleotidase, cytosolic II [Source:HGNC Symbol;Acc:HGNC:8022]" | 9595 | -0.283 | -0.0913 | No |
| 14 | PNP | purine nucleoside phosphorylase [Source:HGNC Symbol;Acc:HGNC:7892] | 9657 | -0.288 | -0.0343 | No |
| 15 | NADK | NAD kinase [Source:HGNC Symbol;Acc:HGNC:29831] | 9949 | -0.316 | 0.0109 | No |
| 16 | NNMT | nicotinamide N-methyltransferase [Source:HGNC Symbol;Acc:HGNC:7861] | 10216 | -0.342 | 0.0637 | No |
| 17 | NAMPT | nicotinamide phosphoribosyltransferase [Source:HGNC Symbol;Acc:HGNC:30092] | 12292 | -0.674 | 0.0472 | No |
Table: GSEA details [plain text format]

  

Fig 2: KEGG\_NICOTINATE\_AND\_NICOTINAMIDE\_METABOLISM      
 Blue-Pink O' Gram in the Space of the Analyzed GeneSet

  

Fig 3: KEGG\_NICOTINATE\_AND\_NICOTINAMIDE\_METABOLISM: Random ES distribution      
 Gene set null distribution of ES for **KEGG\_NICOTINATE\_AND\_NICOTINAMIDE\_METABOLISM**
